# Responses to hypothetical health scenarios overestimate healthcare utilization for common infectious syndromes: a cross-sectional survey, South Africa, 2012

**DOI:** 10.1186/s12879-018-3252-0

**Published:** 2018-07-25

**Authors:** Karen K. Wong, Adam L. Cohen, Neil A. Martinson, Shane A. Norris, Stefano Tempia, Claire von Mollendorf, Sibongile Walaza, Shabir A. Madhi, Meredith L. McMorrow, Cheryl Cohen

**Affiliations:** 1 Centers for Disease Control, 1600 Clifton Rd NE, MS C-09, Atlanta, GA 30329 USA; 2 United States Public Health Service, Rockville, USA; 3 MRC Developmental Pathways for Health Research Unit, University of Witwatersrand, Johannesburg, South Africa; 4 Johns Hopkins University, Baltimore, MD USA; 5 University of Witwatersrand, Johannesburg, South Africa; 6 National Institute for Communicable Diseases, Johannesburg, South Africa

**Keywords:** Pneumonia, Influenza, Diarrhea, Meningitis, South Africa, Healthcare utilization

## Abstract

**Background:**

Asking people how they would seek healthcare in a hypothetical situation can be an efficient way to estimate healthcare utilization, but it is unclear how intended healthcare use corresponds to actual healthcare use.

**Methods:**

We performed a cross-sectional survey between August and September 2012 among households in Soweto and Klerksdorp, South Africa, to compare healthcare seeking behaviors intended for hypothetical common infectious syndromes (pneumonia, influenza-like illness [ILI], chronic respiratory illness, meningitis in persons of any age, and diarrhea in a child < 5 years old) with the self-reported healthcare use among patients with those syndromes.

**Results:**

For most syndromes, the proportion of respondents who intended to seek healthcare at any facility or provider (99–100%) in a hypothetical scenario exceeded the proportion that did seek care (78–100%). More people intended to seek care for a child < 5 years old with diarrhea (186/188 [99%]) than actually did seek care (32/41 [78%], *P* < 0.01). Although most people faced with hypothetical scenarios intended to seek care with licensed medical providers such as hospitals and clinics (97–100%), patients who were ill reported lower use of licensed medical providers (55–95%).

**Conclusions:**

People overestimated their intended healthcare utilization, especially with licensed medical providers, compared with reported healthcare utilization among patients with these illnesses. Studies that measure intended healthcare utilization should consider that actual use of healthcare facilities may be lower than intended use.

## Background

Studying the health seeking behaviors of a community is important to characterize unmet health needs [1, 2], to identify and address barriers to care [3, 4], and to plan healthcare delivery and health education programs [5–8], as well as to adjust estimates of burden of diseases from population-based sentinel surveillance data [9–11]. Healthcare utilization surveys have been conducted to characterize the patterns of health seeking behavior in different communities [1, 5–8, 10–12].

However, these surveys often require large sample sizes to identify enough respondents with the medical problems under investigation and to assess the healthcare seeking behavior related to those problems. A possible solution, that requires fewer resources, would be to ask people how they intend to access healthcare in a hypothetical illness scenario [13–19]. While this approach can be useful for obtaining qualitative data about health knowledge and attitudes towards different healthcare facilities [13, 15, 18], it may not reflect real healthcare utilization by ill people.

In this study, we sought to determine how intended health seeking behaviors in response to hypothetical scenarios compared with actual reported healthcare utilization for common infectious disease syndromes.

## Methods

### Study setting

The study was conducted between August and September 2012 as part of a larger survey about healthcare utilization behaviors at two sites in South Africa: Soweto, an urban township outside Johannesburg, and the peri-urban townships surrounding Klerksdorp, including Jouberton, Alabama, Sakhroi, Kanana, Khuma, Tigane, Dominionville, and Vaal Reefs (Wong KK, von Mollendorf C, Martinson N, Norris S, Tempia S, Walaza S, Variava E, McMorrow ML, Madhi S, Cohen C, Cohen AL: Healthcare utilization for common infectious disease syndromes in Soweto and Klerksdorp, South Africa. forthcoming). Medical care at public healthcare facilities in South Africa is free to children < 18 years old and available for a nominal fee to adults. In addition to public healthcare facilities, people may also seek care with private doctors or facilities, pharmacies, and traditional healers. Soweto is served by a large public secondary-tertiary care hospital (Chris Hani Baragwanath Academic Hospital [CHBAH]), and residents also access other clinics, private general practitioners, and other providers. Adults who present to CHBAH with non-urgent issues are referred to a primary care clinic. Residents of the Klerksdorp site townships are served by the public Klerksdorp/Tshepong Hospital Complex, which is a referral hospital. Hospital emergency services are reserved for emergent issues; non-urgent issues are referred to a primary care clinic. Interviews were conducted in the preferred language of the household and included English, Xhosa, Setswana, and Zulu.

### Study design

We conducted a cross-sectional survey of households at each of the two study sites using a one-stage cluster design similar to methods used by Lindblade et al. [12]. Briefly, random geographic coordinates were generated within residential areas. The sample size was created to estimate healthcare utilization for pneumonia, assuming incidence of 2% per year and 50% prevalence of seeking healthcare for pneumonia of any severity, with 95% confidence intervals and 10% precision. Pneumonia was selected when calculating sample size because the main healthcare utilization survey was designed to complement existing sentinel surveillance for severe acute respiratory illness. Teams visited the household nearest to the geographic coordinate up to three times on separate days to interview household members about recent episodes of illness. The person who had been ill was asked to provide information about the healthcare sought for that illness; if the person was unavailable or was a child, the self-identified primary caregiver was asked to respond on that person’s behalf. We examined illnesses meeting criteria for the following common infectious syndromes:

- Pneumonia within the last year diagnosed by a healthcare worker, or defined by sudden onset or worsening fever (temperature [T] > 38 °C or subjective) and cough and difficult breathing lasting 2–30 days, in a person of any age
- Influenza-like illness [ILI] within the last 30 days defined as sudden onset fever or worsening fever (*T* > 38 °C or subjective) with cough and/or sore throat in a person of any age
- Chronic febrile respiratory illness within the last year defined by fever and cough and either difficult breathing or weight loss lasting ≥30 days in a person of any age
- Diarrhea within the last 14 days defined by ≥3 loose or watery stools within a 24 h period in a child < 5 years of age
- Meningitis in a person of any age within the last year, defined by fever or headache, and one of the following: stiff neck, confusion, new weakness in arm or leg, or double vision.

Case definitions for pneumonia, ILI, and diarrhea were based on those used in other healthcare utilization surveys [9, 12, 20–23]. The criteria for chronic febrile respiratory illness were selected to be consistent with symptom screening tests for tuberculosis [24]. The meningitis case definition was designed by expert consensus to be sensitive for syndromes consistent with either acute or chronic meningitis. In households where a member met criteria for an infectious disease syndrome, we asked about the first place he or she sought care during the illness. Licensed medical providers were defined as any of the following: public clinics (including health centers), public hospitals, private clinics, private hospitals, or private general practitioners.

In addition to the survey on actual healthcare seeking, a random subsample of households (each household independently had a 1/10 probability of selection) at each of the study sites was asked about intended healthcare use for hypothetical scenarios in which a member of their household exhibited symptoms of one of the five common infectious syndromes above. The questionnaire about hypothetical healthcare seeking was performed after the main survey. It was possible but not common for a household to report real illness and also be randomly selected for the hypothetical healthcare seeking survey. For each infectious syndrome, responses about hypothetical healthcare seeking were excluded from the analysis if a real episode of the same syndrome had been reported in the household. The hypothetical infectious syndromes were presented as:

- Pneumonia: Sudden onset or worsening fever and difficult or fast breathing
- ILI: Sudden onset or worsening fever with cough or sore throat
- Chronic febrile respiratory illness: Cough with fever or weight loss lasting > 30 days
- Diarrhea in a child < 5 years old: 3 or more loose stools per day
- Meningitis: Sudden onset of fever and stiff neck

These case definitions were simplified from the case definitions used for actual healthcare seeking to make them easier to understand in the hypothetical setting, but otherwise kept as consistent as possible with the previous case definitions. The interviewer read a list of all possible places to seek care, including not seeking care outside the home at all, before asking the primary caregiver of the household to identify the first place a member of the household would likely seek care in the hypothetical scenario. The case definitions were explained to participants by their symptom criteria to avoid misinterpretation of the syndrome, such as interpreting the term “flu” as a milder upper respiratory infection.

### Statistical analysis

We compared the responses of people presented with a hypothetical illness scenario to the responses about actual healthcare seeking of those not selected for the hypothetical survey. Analysis was conducted using SAS 9.3 (Cary, NC). We used the Chi-square test and Fisher’s exact test to evaluate differences between two or more proportions. Wilcoxon rank-sum test was used to evaluate differences in medians.

## Results

For the main survey of actual healthcare seeking for recent episodes of infectious syndromes, 3382 random geographic coordinates were generated, of which 248 (7%) were not located near a residence. Of 3134 households visited, 258 (8%) declined participation, and for 461 (15%), the primary caregiver was unavailable. There were 2415 (77%) households that completed the survey for actual healthcare seeking. Of these, 1442 (60%) were in Klerksdorp and 973 (40%) were in Soweto. The median age of the primary caregiver was 39 years (interquartile range [IQR]: 29–52), and 1674 (69%) of primary caregivers were female. There were 1438 (68%) households with a monthly income of < ZAR 2000 (< 237 USD as of September 1, 2012) [25]. The households comprised 10,461 individuals. Among household members, there were 262 episodes of pneumonia, 296 episodes of ILI, 22 episodes of chronic respiratory illness, 26 episodes of meningitis, and 52 episodes of diarrhea in a child < 5 years old within the time periods specified in the case definitions.

Of the households visited, 300 (10%) were randomly selected to complete the additional survey; 24 (9%) declined the main and additional surveys, 22 (8%) completed the main survey but declined the additional survey, 41 (15%) did not have a primary caregiver available, and 190 (69%) completed the additional survey. There were no meaningful differences in primary caregiver age, caregiver sex, study site, or monthly income < ZAR 2000 between those who completed the additional survey about hypothetical health scenarios and those who completed the main survey only (Table 1). Lower response rates were noted for the survey about hypothetical compared with actual behaviors, as the hypothetical survey was given after the main survey about actual behaviors, and some participants who completed the main survey declined to complete the additional survey.

**Table 1:** Characteristics of households surveyed about actual healthcare utilization and subset of households surveyed about hypothetical healthcare utilization, South Africa, 2011–2012

	Subset surveyed about hypothetical healthcare utilization	Main survey of healthcare utilization, excluding subset	*P*-value
Number of geographic coordinates sampled, N	300	3082	NA
Number of invalid coordinates, n (%)	23 (8)	225 (7)	n.s.
Households visited, n (%)	277 (92)	2857 (93)	n.s.
Declined main survey, n (%)	24 (9)	234 (8)	n.s.
Declined additional survey only, n (%)	22 (8)	NA	NA
Primary caregiver unavailable, n (%)	41 (15)	420 (15)	n.s.
Completed survey(s), n (%)	190 (69)	2203 (77)	< 0.01
Primary caregiver age, median years (IQR)	37 (28–52)	39 (29–52)	n.s.
Primary caregiver female, n (%)	138 (73)	1536 (70)	n.s.
Site			
Klerksdorp, n (%)	114 (60)	1318 (60)	n.s.
Soweto, n (%)	76 (40)	885 (40)	n.s.
Monthly income <ZAR 2000, n (%)	116 (69)	1307 (68)	n.s.

*NA* Not applicable

Almost all survey respondents reported that a member of the household would seek any healthcare (licensed medical providers or other providers or facilities) if he or she were ill in the hypothetical scenarios (99–100%) (Table 2). Among participants who met criteria for any of the infectious syndromes, the proportion who actually sought any care for the episode of illness varied. Only 32/41 (78%) ill children < 5 years old who had diarrhea were taken to seek any care; however, 180/182 (99%) households presented with a hypothetical scenario of diarrhea in a child < 5 years old said that they would seek care, and 97% would seek care with a licensed medical provider. Prevalence of actual healthcare use was 79% (213/268) among patients with ILI and 85% (197/231) among those with pneumonia compared with 99–100% for both hypothetical scenarios (*P* < 0.05). Almost all survey respondents reported the intention to seek care with a licensed medical provider first (97–100%) for hypothetical illness scenarios. However, the proportion of patients who reported an actual initial consultation with a licensed medical provider was 55% (147/268) among ILI patients. The proportions of consultations with a licensed medical provider for actual illnesses was consistently lower compared with hypothetical illnesses. The proportion that consulted a pharmacy first was highest for ILI (59/268 [22%]) and pneumonia (43/231 [19%]) patients. Initial consultation with traditional healers, religious leaders, friends or relatives, or health volunteers was infrequent (0–5%) for actual illnesses, and these providers were almost never mentioned as the first provider consulted in hypothetical scenarios (0–1%).

**Table 2:** Intended healthcare utilization among survey respondents^a^ versus actual healthcare utilization among patients for common infectious syndromes — South Africa, 2012

First provider consulted for illness	ILI			Pneumonia			Chronic respiratory			Meningitis			Diarrhea in child <5y		
n (%)^b^	Intended, *n* = 172	Actual, *n* = 268	*P*	Intended, *n* = 172	Actual, *n* = 231	*P*	Intended, *n* = 187	Actual, *n* = 16	*P*	Intended, *n* = 186	Actual, *n* = 21	*P*	Intended, *n* = 182	Actual, *n* = 41	*P*
Would not /did not seek care	(0)	55 (21)	<.01	0	34 (15)	<.01	0	1 (6)	ns	1 (1)	0	ns	2 (1)	9 (22)	ns
Would seek / sought care	170 (99)	213 (79)	<.01	172 (100)	197 (85)	<.01	187 (100)	15 (94)	0.01	185 (99)	21 (100)	ns	180 (99)	32 (78)	<.01
Licensed medical provider	168 (98)	147 (55)	<.01	171 (99)	151 (65)	<.01	187 (100)	15 (94)	0.01	181 (97)	20 (95)	ns	177 (97)	27 (66)	<.01
Public clinic or health center	133 (77)	90 (34)	<.01	122 (71)	98 (42)	<.01	139 (74)	9 (56)	0.1	133 (72)	12 (57)	ns	146 (80)	20 (49)	0.06
Private clinic	19 (11)	9 (3)	<.01	19 (11)	8 (3)	<.01	14 (7)	0	ns	18 (10)	0	ns	17 (9)	2 (5)	<.01
Public hospital	11 (6)	5 (2)	ns	24 (14)	14 (6)	<.01	28 (15)	5 (31)	0.1	21 (11)	7 (33)	0.01	9 (5)	0	ns
Private hospital	3 (2)	9 (3)	ns	4 (2)	7 (3)	ns	4 (2)	0	1	6 (3)	0	ns	2 (1)	0	ns
Private Doctor	2 (1)	34 (13)	<.01	2 (1)	24 (10)	<.01	2 (1)	1 (6)	ns	3 (2)	1 (5)	ns	3 (2)	5 (12)	ns
Other provider	2 (1)	66 (25)	<.01	1 (1)	46 (20)	<.01	0 (0)	0	0.01	4 (2)	1 (5)	ns	3 (2)	5 (12)	0.007
Pharmacy	2 (1)	59 (22)	<.01	(0)	43 (19)	<.01	(0)	0	0.01	3 (2)	0	ns	3 (2)	2 (5)	0.007
Traditional healer	(0)	1	ns	1 (1)	1	ns	(0)	0	0.1	(0)	0	ns	(0)	1 (2)	ns
Religious leader	(0)	0	ns	(0)	1	ns	(0)	0	ns	(0)	0	ns	(0)	0	ns
Friend or relative	(0)	4 (1)	ns	(0)	0	ns	(0)	0	0.1	(0)	1 (5)	ns	(0)	1 (2)	ns
Health volunteer	(0)	1	ns	(0)	1	ns	(0)	0	1	(0)	0	ns	(0)	0	ns
Other	(0)	1	ns	(0)	0	ns	(0)	0	ns	1 (1)	0	ns	(0)	1 (2)	ns

^a^For each infectious syndrome, respondents who reported an actual episode of the infectious syndrome within the household were excluded from the analysis of intended healthcare utilization

^b^Excludes episodes of ILI (1), pneumonia (4), chronic respiratory illness (1), meningitis (1), and diarrhea (1) for which the first provider consulted was unknown

*P*-values greater than 0.1 are indicated with “ns”

## Discussion

Responses to hypothetical scenarios overestimated the actual utilization of healthcare for common infectious disease syndromes. While most participants anticipated consulting licensed medical providers first for symptoms in a hypothetical scenario, ill patients often did not seek care with licensed medical providers for their actual illnesses, or they first visited other facilities, such as pharmacies.

Results from prior studies comparing hypothetical and actual healthcare utilization are mixed. In contrast to our findings, studies conducted in Vietnam [26] and Mexico [14] revealed no meaningful difference between hypothetical and actual health seeking behaviors for diarrhea and dysentery; the authors of one study used this finding to justify combining the actual and hypothetical cases in order to analyze the healthcare preferences of the population [26]. In a study among Indonesian mothers, seeking care for a hypothetical infant with signs of severe pneumonia approximated actual care-seeking in some analyses but not in others [27]. The mixed conclusions of these studies may be due to differences in study methods and the hypothetical scenarios presented, or they may reflect differences in behavior between populations. Other studies have found that hypothetical healthcare utilization overestimates actual healthcare utilization, as seen in our study. A study conducted in Southeast Nigeria about preferences for treatment of malaria revealed that surveys about hypothetical healthcare use overestimated the proportion that would seek care at public health facilities and showed that people were more likely to self-treat at home [28]. Similarly, a study of healthcare utilization for stroke in the United States revealed that far more people intended to use emergency services for hypothetical stroke symptoms than actually did use them when real symptoms occurred [29].

This study has certain limitations. For both hypothetical and actual healthcare utilization, analysis was limited to the first place consulted for the illness. Asking participants to list multiple providers or to rank providers in order of consultation may have produced different results. Primary caregivers were the respondents for the hypothetical scenarios, and they may be likely to think of their own behavior or what they would do for children in the household when imagining the health scenarios, rather than anticipate the behavior of other adult household members. Case definitions for the actual illnesses experienced and the hypothetical infectious syndromes differed slightly in order to make the survey easier to understand, and the differences in wording may have led to different interpretation. When reporting actual health seeking behavior, some misclassification of providers by the respondents may have occurred; for instance, participants may have thought of consulting friends or relatives as equivalent to not seeking care. It is possible that respondents would have reported a high prevalence of healthcare utilization for hypothetical scenarios, especially with licensed medical providers, because it was perceived as being a more socially desirable response [30]. Some respondents may have failed to report episodes of actual healthcare utilization due to poor recall. Recall of healthcare utilization for illnesses may have been imprecise, particularly for milder illnesses and for those requiring a longer recall period such as chronic respiratory illness [31]. It is unclear how severity of illness may have affected bias. For actual illness, people may have been more likely to recall more severe illnesses requiring medical attention; conversely, some respondents may have interpreted hypothetical scenarios as being more severe.

## Conclusions

Although characterizing anticipated healthcare utilization with hypothetical scenarios may be an efficient method of exploring attitudes towards healthcare, in this study, responses to hypothetical scenarios overestimated actual health seeking behaviors of the communities. Those who survey populations on hypothetical healthcare use to plan healthcare delivery or estimate disease burden from surveillance data should consider whether the survey may overestimate actual use for their study population and research question.

## Abbreviation

ILI: influenza-like illness
